# Comparison of the toxicity of aqueous and ethanol fractions of *Angelica keiskei* leaf using the eye irritancy test

**DOI:** 10.3892/etm.2012.677

**Published:** 2012-08-20

**Authors:** HYEONG-U SON, EUN-KYUNG YOON, YONG-SOO CHA, MIN-A KIM, YONG-KYU SHIN, JONG-MYUNG KIM, YONG-HEE CHOI, SANG-HAN LEE

**Affiliations:** 1 Department of Food Science and Biotechnology, Graduate School, Kyungpook National University, Daegu 702-701;; 2 Bion Co. Ltd., Techno Building, Daegu 702-842;; 3 Food and Bio-Industry Research Institute, Kyungpook National University, Daegu 702-701, Republic of Korea

**Keywords:** *Angelica keiskei*, eye irritancy test, fraction, cosmetic ingredient

## Abstract

To determine whether aqueous and ethanol fractions of the *Angelica keiskei* leaf exert toxicity when used for cosmetic purposes, we performed the acute eye irritancy test. Animals were treated with sample fractions (100 mg/dose) according to standard procedure guidelines. No significant changes or damage was detected in the fraction-treated groups in terms of ocular lesions in the cornea, the size of the cornea with turbidity, swelling of the eyelid and emission discharge. However, sodium dioctyl sulfosuccinate, a positive control, induced severe toxic symptoms. Thus, aqueous and ethanol fractions of *Angelica keiskei* do not appear to induce acute toxicity in the eye lens, as assessed from anatomical and pathological observations in the rabbit eye. Our results collectively suggest that aqueous and ethanol fractions show promise as cosmetic ingredients that do not cause eye toxicity.

## Introduction

Many medicinal plants provide beneficial sources of vitamins, dietary fiber and phytochemicals. A number of herbs are widely used owing to their flavor and healthy constituents which, not only regulate body homeostasis, but also prevent degenerative diseases (1–3). *Angelica* sp. is a common vegetable known for its vitamin content (4). Early studies have shown that the vegetable contains vitamins C, B1 and B2, chlorophylls and diverse minerals (4,5). The moist leaves exhibit exceptional antioxidant activity and therefore the herb is widely used as a functional food ingredient. Additionally, the herb has therapeutic effects on hypertension, constipation, diuresis, arteriosclerosis and most importantly cancer, which are attributable to its small molecular constituents, such as flavonoids, saponin and coumarins (5). Further research on the chemical composition of oils has led to the identification of dozens of small compounds (6). The oils exhibit strong activity in suppressing PANC-1 pancreatic cancer cell and Crl breast cancer cell growth (7). *Angelica keiskei* is broadly employed in alternative medicine as a remedy to treat bowel disturbance syndrome, arthritis and immune diseases (8). However, there is limited scientific information regarding its effects on various degenerative disorders. Findings of another study have shown that *Angelica keiskei* has the ability to reduce inflammation in a chronic ethanol-induced *in vivo* test. Upon treatment of ICR mice with 10, 25 and 50 mg/kg extracts p.o., alcohol-induced hepatotoxicity was improved, indicating that *Angelica keiskei* indirectly safeguards the liver against oxidative stress induced by free radicals (9). An earlier investigation by our group revealed anti-asthmatic activities of an aqueous leaf extract in an ovalbumin-induced animal model (10). Mice sensitized to ovalbumin were orally administered the *Angelica archangelica* extract and their lungs were analyzed via hematoxylin and eosin (H&E) staining to measure the IL-4/13 cytokine content. The extract exhibited strong anti-asthmatic activity via regulation of the CD4^+^ cell population, IL-4/13 expression and asthma-related molecular markers in the lungs (10).

The Korea Food and Drug Administration has recommended submission of data from eye irritancy, skin irritancy and phototoxicity tests to obtain approval and authorization of the use of test compounds as functional cosmetic ingredients(s). Plant extracts with pungent flavors appear to cause irritation when exposed to the skin (4). Unwanted mild or transient reactions to cosmetics are common in patients with allergic contact dermatitis. Various adverse effects including acute/chronic toxicity, irritation and sensitization, can be assessed using *in vivo*, *in vitro*, semi *in vivo* and *ex vivo* animal models, even in modified tests (11–13). A specific component or constituent should not exert toxic effects on the skin (particularly the eye in the case of cosmetics) and should only be passed and approved in cases where no damage/changes to the eye lens are observed in animals or clinical trials for the development of cosmetics (14). Although cosmetic ingredients rarely trigger serious damage, their advantages in skin protection or tissue regeneration should be emphasized.

In the current study, we performed the acute eye lens mucosal irritation test with *Angelica keiskei* leaf fractions using an *in vivo* animal model. Various parameters were assessed by comparing the degree of acute toxicity induced to confirm whether these fractions have potential for development in cosmetic applications.

## Materials and methods

### Animals and care

New Zealand White rabbits (9 weeks old, male, weighing 2.0–2.2 kg) were purchased from M&J Animal Supplies Co. (Seoul, Korea) and fed a commercial diet (Purina, Seoul, Korea) and water *ad libitum*. Animal protocols were performed in accordance with the guidelines of the Committee of International Association for the Study of Pain on Research and Ethical Issues (15). The rabbits were allowed to adjust to the laboratory surroundings for at least 1 week prior to the experiments. The number of rabbits in each group was 3.

### Sample preparation

**Figure 1:**
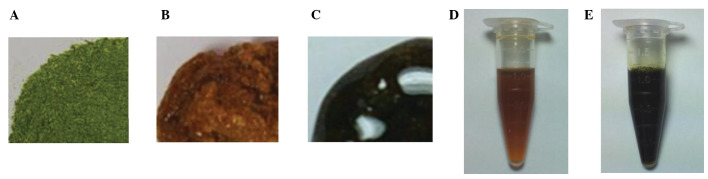
Images of the aqueous and ethanol fractions of *Angelica keiskei* leaf are shown. (A) Slice-dried leaf was pulverized and extracted with (B) distilled water or (C) ethanol at 60°C for 12 h in a shaking incubator and filtered. Lyophilization was performed to obtain the final sample powder. The (D) aqueous and (E) ethanol fractions (each 100 mg/ml) are shown.

*Angelica keiskei* leaf purchased from Myung-il Farm Co. (Eumsung, Korea) was used throughout the experiments. The slice-dried leaf was pulverized with a homogenizer (20,000 rpm for 15 min; Shinil, Seoul, Korea). Powder was extracted with distilled water (powder:distilled water = 1:10; w/v) or ethanol (powder:50% ethanol = 1:6; w/v) at 60°C for 12 h in a shaking incubator (JSR, Gongju, Korea), followed by filtration (Filter paper No. 1, Whatman, Schleicher & Schuell, Buckinghamshire, UK). Lyophilization was carried out in a freeze-dryer (Bondiro, Il-Shin, Seoul, Korea) to obtain the final samples (Fig. 1B and C). The voucher specimens of the *Angelica keiskei* leaf and powder were deposited in the Laboratory of Food Enzyme Biotechnology, Kyungpook National University (#2010-Ak).

### Draize eye irritancy test

Fractions (100 mg/ml of concentrated aqueous and ethanol fractions) were dripped into the eyes of each New Zealand White rabbit (n=5) with their eyes held open and clipped at the lid. As a positive control, 10% of sodium dioctyl sulfosuccinate was employed. Progressive damage/changes to the rabbit eye were recorded each day for 7 days. Reactions to the fractions included swelling of the eyelid, iris inflammation, ulceration and hemorrhaging, as described in other studies, with slight modifications (16,17).

### Sample treatment

Following instillation of the aqueous and ethanol fractions of the *Angelica keiskei* leaf, eye lens mucosa was evaluated for local mucosal irritation. Saline was used as the control. The conjunctival sac in the right eye of the rabbits was treated with the sample (Fig. 1D and E; concentrated to 0.1 ml), control (saline) or positive control (sodium dioctyl sulfosuccinate). After applying once for 2 sec, washing with saline was performed as for the control group. The undiluted sample was administered once under the eyelid, which was slightly pulled away to form a space to allow easy administration (aqueous or ethanol fraction, 0.1 ml each) into the conjunctival sac. Following this, the cornea, iris and conjunctiva were examined daily at the designated times (1, 2, 3, 4, 5, and 7 days) to evaluate acute toxicity in the eye lens mucosa.

### Analysis of parameters

Lesions were observed in experiments whereby the test substance was not applied to the left eye for comparison purposes. On days 1, 2, 3, 4, 5, and 7 following the application of the test fractions, we examined the following criteria of the naked eye: corneal opacity, turbidity range, reaction of the iris and conjunctiva, edema and emission discharge. Irritancy in the eye lens mucosa was evaluated based on redness, irritancy, ocular lesions or inflammation by trained examiners under the authority of a professional from the Center of Laboratory Animal and Care, Kyungpook National University.

## Results and Discussion

In the course of screening natural resources such as food and/or oriental herb plants for active components exerting anti-inflammatory effects in cosmetic application, we observed that aqueous and ethanol fractions of the *Angelica keiskei* leaf exhibit a potent whitening effect at a concentration of 100 μg/ml with no cytotoxicity in both *in vitro* and *in vivo* assays (data not shown).

Recently, *Angelica* sp. has become a valuable food source in Asia, as these edible plants provide health benefits owing to their antioxidant activities, although some species have a pungent flavor (4). The leaf is consumed by vegetarians both in restaurants and at home and contains various nutrients including vitamins, flavonoids, flavonol and other polyphenol compounds (9,10). For the approval of cosmetics produced using food or chemical sources, the Korean Food and Drug Administration accepts only safety data derived from the most widely used animal tests, such as the Draize eye irritancy test, which involves placing drops of the substance into rabbit eyes. The guidelines for the use of a test compound in cosmetics as a mixture/ingredient involves the control of specific skin toxicity (18). The main constituents associated with toxicity are complicated by the presence of various components such as amine, nitrous compounds and other beneficial and/or disadvantageous substances. Nevertheless, several reports on the antitumor, anti-diabetic and anti-inflammatory activities of known or unknown extracts/fractions of herbal or medicinal plants have been documented thus far (2,3). In previous studies aimed at promoting biological and other applicable purposes, we examined whether these fractions/extracts have biological activity in ameliorating degenerative disorders, including atopic dermatitis. Initially, food ingredients are produced using raw grains, cereals, fruits, vegetables and medicinal herbs and thereafter processed into biomaterials using a range of methods. Following the application of valuable supplementary techniques, such as supercritical extraction, microbial fermentation, biotransformation or chemical modification, biomaterials may be converted into a cosmetic, cosmeceutical, neutraceutical or drug. For this reason, we developed a screening system for antioxidant and anti-tyrosinase agents from medicinal plant extracts and examined their anti-asthmatic activities, as described previously (19,20).

Safety data were obtained following the administration of aqueous and ethanol fractions of the *Angelica keiskei* leaf (Fig. 1D and E; 100 mg/ml in a total volume of 100 μl) into rabbit eyes. When saline was used as the control, we did not observe congestion symptoms around the pupil and whites of the eye (data not shown). The Draize eye irritancy test is strictly observational and does not adequately reflect the degree of irritancy in humans, therefore it is generally considered crude, imprecise and unreliable. However, this remains the most convincing test in animal models. We applied the following criteria for precise evaluation of toxic symptoms: swelling, inflammation and ulceration on the eye lens. Initially, following treatment with aqueous and ethanol fractions or saline, eyelid and eye mucosa membranes were examined daily. In terms of ocular lesions in the cornea, scores were measured as described in Materials and methods. Lesions were scored using a point system: 0 for no suppuration or haze, 1 when slightly opaque compared to the normal difference in transparency, 2 when easily observed as partly semi-transparent, 3 when not observed at the end of the pupil and 4 when the cornea, but not the iris, was opaque and turbid.

**Figure 2:**
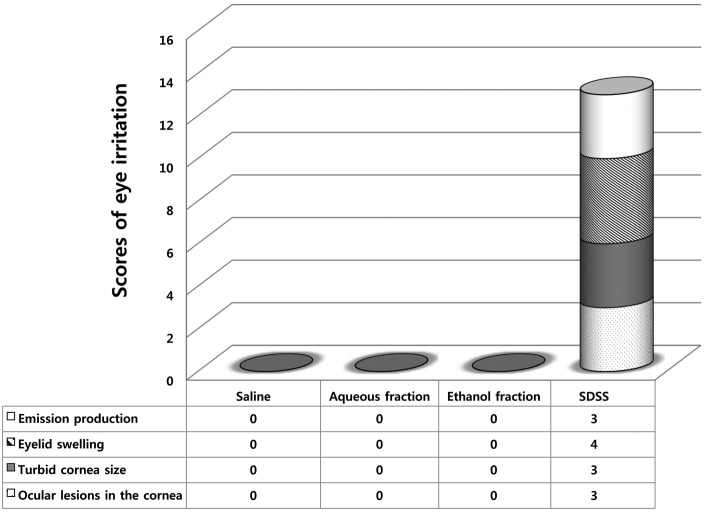
Comparison of the aqueous and ethanol fractions of the *Angelica keiskei* leaf in an acute eye lens mucosal irritancy test. Final scores of acute eye lens mucosal irritancy are shown. Each score is the sum of the total degree. Data denote a classical result of five independent observations. Dotted, ocular lesions in the cornea; black, turbid cornea size; slashed, eyelid swelling; white, emission production. SDSS, sodium dioctyl sulfosuccinate.

Aqueous and ethanol fractions of *Angelica keiskei* leaf did not induce perforated ocular lesions in the cornea (Fig. 2, dotted section of column 4), similar to the patterns observed with saline (Fig. 2, column 1) with no additional symptoms. In terms of turbid cornea size, scores were measured as follows: 0 for no turbidity, 1 when the size of the cornea was 1/4 or less, 2 when greater than 1/4 but less than 1/2, 3 when greater than 1/2 but less than 3/4 and 4 when greater than 3/4 up to a value of 1 or more. Notably, cornea size and turbidity were not affected by the fractions (Fig. 2, columns 3 and 4). Additionally, the effects of the fractions on eyelid swelling were examined. Eyelid swelling was scored as follows: 0 for no swelling, 1 when normal or slightly swollen (including nictitating membrane), 2 in cases of significant swelling of the eyelid resulting in partial abduction, 3 when swelling of the eyelid was approximately half of the wound and 4 when more than half of the eyelid was swollen. Using the point scoring system, we confirmed that the eyelid was not affected in terms of swelling upon treatment with the fractions (Fig. 2, columns 2 and 3). Production of emissions was analyzed as follows: 0 for no emission, 1 when a small amount of internal eyelash was observed, 2 for wet exhaust and 3 when a large area around the eye and eyelid and/or eyelashes had wet exhaust. As a result, no emission was observed in the eye or eyelid/eyelashes following treatment with the fractions (Fig. 2, columns 2 and 3).

**Figure 3:**
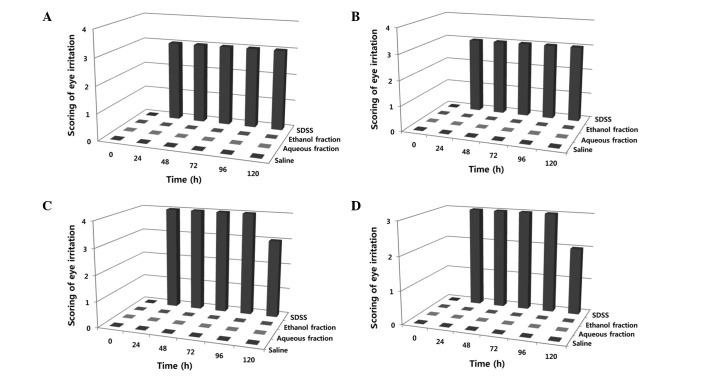
Observation of acute eye irritancy at set time intervals. Scoring standards are similar to those presented in Materials and methods. Observations were made at 6 time-points: 0, 24, 48, 72, 96, and 120 h. The scores of eye irritation are shown at each time interval. (A) Ocular lesions in the cornea, (B) turbid cornea size, (C) eyelid swelling and (D) emission production. Data denote a classical result of five independent observations. Saline, 1st lanes; aqueous fraction, 2nd lanes; ethanol fraction, 3rd lanes; SDSS, 4th lanes. SDSS, sodium dioctyl sulfosuccinate.

Overall, we detected no changes or damage induced by either the aqueous or ethanol fractions in contrast to sodium dioctyl sulfosuccinate which caused severe toxic symptoms (Fig. 2, comparing column 4 with columns 2 and 3), as assessed by anatomical and clinical observations. The criteria for determining whether or not other parameters are associated with acute eye irritancy were assessed. Observations at the set time intervals (day 1, 2, 3, 4 and 5) revealed that neither the aqueous nor the ethanol fractions affected the ocular lesions in the cornea (Fig. 3A, lanes 2 and 3), turbid cornea size (Fig. 3B, lanes 2 and 3), eyelid swelling (Fig. 3C, lanes 2 and 3) and emission production (Fig. 3D, lanes 2 and 3), whereas sodium dioctyl sulfosuccinate induced perforated damages (Fig. 3, lane 4 of A, B, C and D). Additionally, we did not specify two recommended tests in this report; however, skin irritancy and phototoxicity tests were also negative, whereby wounds treated with the fractions showed similar patterns of recovery as those treated with the control (PBS), and UV exposure of 8-methoxypsoralen plus fraction treatments did not induce tumors in mouse skin (data not shown). A few positive toxicity cases have reportedly been identified from other safety tests, and therefore, we cannot exclude the possibility of toxicity based on acute, sub-acute or chronic safety tests, such as the soap chamber test, repeat insult test or murine local lymph node assay (21–23).

In summary, *Angelica keiskei* leaf fractions do not induce acute toxicity in the eye irritancy test; specifically, no haze, swelling, redness or emissions in the eye mucosa were observed. *Angelica keiskei is* therefore a potential candidate for development in the cosmetic industry and/or other applicable purposes. We await the purification and isolation of the pure compound(s), which should aid in establishing those that may be successfully applied for long-term use with no hazardous effects, such as phytoestrogens and toxicants.
